# Attribution of dispersal limitation can better explain the assembly patterns of plant microbiota

**DOI:** 10.3389/fpls.2023.1168760

**Published:** 2023-10-24

**Authors:** Taiqiang Li, Jiangyun Gao

**Affiliations:** Institute of Biodiversity, School of Ecology and Environmental Science, Yunnan University, Kunming, China

**Keywords:** endophytic root microbiota, community assembly, host filtering effects, active migration, agricultural sustainability

## Abstract

Disentangling community assembly processes is crucial for fully understanding the function of microbiota in agricultural ecosystems. However, numerous plant microbiome surveys have gradually revealed that stochastic processes dominate the assembly of the endophytic root microbiota in conflict with strong host filtering effects, which is an important issue. Resolving such conflicts or inconsistencies will not only help accurately predict the composition and structure of the root endophytic microbiota and its driving mechanisms, but also provide important guidance on the correlation between the relative importance of deterministic and stochastic processes in the assembly of the root endophytic microbiota, and crop productivity and nutritional quality. Here, we propose that the inappropriate division of dispersal limitation may be the main reason for such inconsistency, which can be resolved after the proportion of dispersal limitation is incorporated into the deterministic processes. The rationality of this adjustment under the framework of the formation of a holobiont between the microbiome and the plant host is herein explained, and a potential theoretical framework for dynamic assembly patterns of endophytic microbiota along the soil–plant continuum is proposed. Considering that the assembly of root endophytic microbiota is complicated, we suggest caution and level-by-level verification from deterministic processes to neutral components to stochastic processes when deciding on the attribution of dispersal limitation in the future to promote the expansion and application of microbiome engineering in sustainable agricultural development based on community assembly patterns.

## Introduction

Endophytic microbes have attracted significant attention owing their great potential to support plant fitness and sustainable agricultural production (Trivedi et al., 2020; Bai et al., 2022; Giovannetti et al., 2023); thus, community assembly mechanisms have long been the focus of microbial ecology research. Using the immune system, secretions, and genetic networks, plants dynamically recruit or inherit taxonomically and functionally diverse endophytic microbial taxa from the soil, seeds, and environment via vertical or horizontal transmission (Cordovez et al., 2019; Fitzpatrick et al., 2020; Bai et al., 2022). This assembly process is concomitantly affected by the host, environment, interactions between microbes, and dispersal and is primarily driven by compartmentalization and developmental stages, which substantially promote the evolution of symbiotic cooperation (Trivedi et al., 2020; Xiong et al., 2021a; Xiong et al., 2021b; Santoyo, 2022). Furthermore, host effects on microbial communities exhibit a gradually increasing trend from soils (bulk soil and rhizosphere) to plant external tissues (rhizoplane and phylloplane) to plant internal tissues (root endosphere and leaf endosphere) (Xiong et al., 2021a). To date, two community assembly models have been proposed for the recruitment of root endophytic microbiota (Edwards et al., 2015; Wang et al., 2020). Briefly, a two-step or multi-step model indicates that endophytic microbial colonization in the roots results from the gradual enrichment or depletion of specific microbial assemblages in the bulk soil (Edwards et al., 2015; Maciá-Vicente et al., 2020). In contrast, the amplification-selection model indicates that microbial communities in the bulk soil (in the countryside/desert) first experience substantial enrichment in the rhizosphere soil (in the metropolis/oasis; implying that they have more available resources) during root recruitment of endophytic microbiota and then some specific taxa are highly selected by the host to successfully colonize the root (Wang et al., 2020; Marasco et al., 2022).

**Figure 1:**
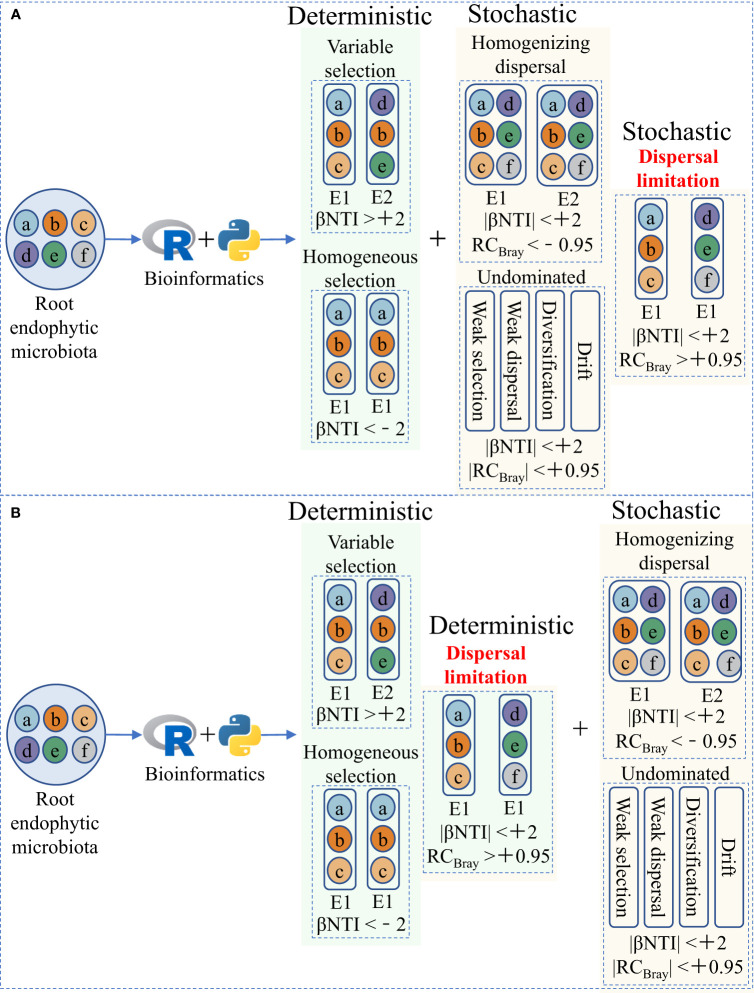
Two ecological null modeling frameworks for quantitative microbial community assembly. **(A)** The current widely accepted and applied microbial community assembly framework, modified from Stegen et al. (2013) and Zhou and Ning (2017). **(B)** The microbial community assembly framework we hypothesize in this article, in which dispersal limitation is incorporated into deterministic processes under strong host selection pressure, can better explain the assembly of root endophytic microbiota. E1 and E2 represent two different environments. βNTI, the β-nearest taxon index, represents the phylogenetic β-diversity metric. RC_Bray_, the Bray–Curtis-based Raup–Crick, represents the taxonomic β-diversity metric.

Explaining microbiota assembly patterns in different habitats using deterministic and stochastic processes (two potential mechanisms of microbial community assembly) based on the niche and neutral theories, respectively, has been widely accepted and applied in microbial ecology (**Figure 1**; **Table 1**) (Stegen et al., 2013; Ning et al., 2020). Briefly, deterministic processes, such as variable and homogeneous selection, emphasize that successful colonization by microorganisms is driven by competition and interaction, and that microbial community structure depends on biotic and abiotic factors. In contrast, stochastic processes, such as homogenizing dispersal, dispersal limitation, and undominated processes, consider that random changes shape microbial communities and that their fluctuations are random, including unpredictable interference, random birth and death, and dispersal probability (**Table 1**; Stegen et al., 2013; Dini-Andreote et al., 2015; Zhou and Ning, 2017). By establishing and modifying the aforementioned community assembly framework (**Figure 1**), we can better understand plant–microorganism coevolution and develop strategies for targeted manipulation of beneficial microorganisms.

**Table 1:** Key terms used in this article.

Term	Definition	Reference(s)
Microbiota assembly	The process by which microorganisms form communities and maintain specific spatiotemporal diversity, structure, succession, and biogeographic patterns through immigration, colonization, and interaction.	Zhou and Ning, 2017; Cordovez et al., 2019
Ecological null model analysis	Estimating the standard deviation of the phylogenetic β-diversity of the observed microbiota compared to the phylogenetic β-diversity of the randomly shuffled microbiota. If the observed phylogenetic β-diversity is not significantly different from the null expectation, the community assembly process is largely considered to be stochastic; otherwise, community dynamics are explained by deterministic processes.	Chase and Myers, 2011
Deterministic processes	The composition and structure of microbial communities are the result of ecological selection, shaped by interspecific interactions (e.g., competition, mutualism, predation, and trade-offs) and environmental filtering (e.g., soil, climate, and geography).	Vellend, 2010
Variable selection	Phylogenetic turnover or similarity between communities shaped by biotic and abiotic factors is significantly higher than the null expectation.	Stegen et al., 2013; Zhou and Ning, 2017
Homogeneous selection	Phylogenetic turnover or similarity between communities shaped by biotic and abiotic factors is significantly lower than the null expectation.	Stegen et al., 2013; Zhou and Ning, 2017
Stochastic processes	The composition and structure of microbial communities are not associated with environmentally determined fitness and are the result of randomness (including random birth, death, dispersal, speciation, and extinction). All microorganisms are ecologically equivalent.	Hubbell, 2001; Chase and Myers, 2011
Dispersal limitation	Very low rates of dispersal between communities or habitats resulted in their distinctly different structures.	Stegen et al., 2013; Zhou and Ning, 2017
Homogenizing dispersal	Very high rates of dispersal between communities or habitats result in their very similar structures.	Stegen et al., 2013; Zhou and Ning, 2017
Undominated processes	Microbiota assembly is not dominated by any single process, including weak selection, weak dispersal, diversification (mutation), and drift (random fluctuations in microbial abundance).	Stegen et al., 2013; Zhou and Ning, 2017
Host filtering effects	The degree of host influence on microbial communities, which is related to host identity, host phylogenetic distance, and host functional traits.	Maciá-Vicente et al., 2020; Yang et al., 2023
Soil–plant continuum	The microhabitats involved range from soil to plant roots and aboveground parts, including bulk soil, rhizosphere, rhizoplane, root endosphere, caulosphere, stem endosphere, phylloplane and leaf endosphere, among others. Microbial communities along the soil–plant continuum have distinctly different compositions, diversity, and functions (i.e., compartmentalization).	Xiong and Lu, 2022

## Potential ecological functions of plant microbiota under different assembly patterns

Microbiota assembly patterns manifest as large spatial and temporal joint variations driven by selection and random factors throughout the life cycle of plants, which significantly affect plant growth and health (Xiong and Lu, 2022). Increasing evidence has shown that host characteristics and environmental conditions affect crop output and nutritional quality by regulating the ratio of deterministic and stochastic processes (Beschoren da Costa et al., 2022; Zhang et al., 2022). In particular, among the ecological functions that are affected by these processes are: 1) maintenance and creation of microbial diversity and community structure at different spatiotemporal scales, and sensitive responses to environmental disturbances (Gao et al., 2020; Wang et al., 2021; Bell et al., 2022); 2) promotion of species coexistence, and convergence and divergence of functional traits in different ecosystems (Liu et al., 2021a); 3) control of plant diseases (Liu et al., 2022); and 4) increase the number of interactions (i.e., connectivity) in microbial co-occurrence networks (the correct outcome of network construction should be an empty network when the community assembly pattern is completely dominated by stochastic processes) (Faust, 2021). Hence, because the microbiota assembly patterns drive important ecosystem service functions, the accurate division of stochastic and deterministic processes during plant microbiota assembly can help acquire a comprehensive understanding of the associations between plants, microbiota, environment, and agricultural productivity. Taken together, such knowledge can help improve commercially relevant crops, as well as protect rare and endangered plants via potential ecological reciprocal connections.

## Assembly processes inconsistency of root endophytic microbiota due to strong host filtering effects

**Figure 2:**
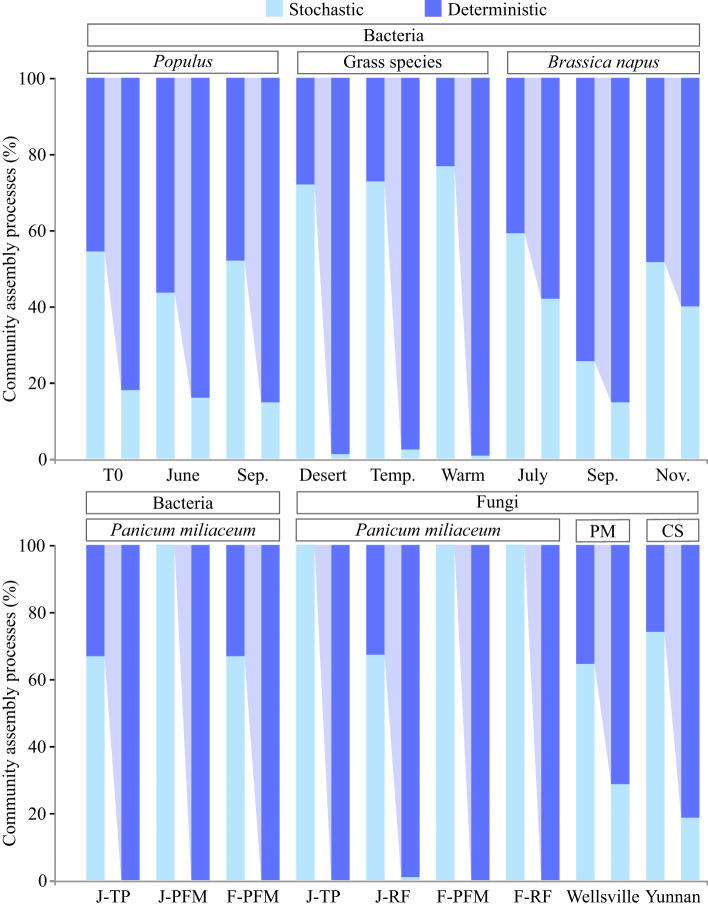
Dispersal limitation is a key factor that determines the relative importance of the deterministic and stochastic processes in the assembly of root endophytic microbiota. The left and right bars corresponding to each treatment represent the relative contributions of the deterministic and stochastic processes before and after dispersal limitation is incorporated into deterministic processes, respectively. Detailed information on each treatment group can be found in the original literature cited. *Brassica napus* (Bell et al., 2022), grass species (Zhong et al., 2022), *Panicum miliaceum* (Tian et al., 2022); *Pseudotsuga menziesii* (PM) (Birch et al., 2022), *Populus* (Dove et al., 2021), and *Camellia sinensis* (CS; unpublished data).

Host selection imposed by the morphology, structure, and function of the root strongly influences the composition, structure, and assembly dynamics of the root microbiota (Herms et al., 2022; Xiong and Lu, 2022). In this context, microorganisms that successfully colonize the roots undergo rigorous host screening, predominantly driven by biotic factors, and usually exhibit significant distance decay patterns (i.e., the spatial pattern in which community similarity decreases as geographical distance increases) (Aas et al., 2019; Fitzpatrick et al., 2020; Roux et al., 2023). Meanwhile, the root endophytic microbiota exhibited a relatively conservative community structure under strong host selection pressure (Wippel et al., 2021; Bourceret et al., 2022). In contrast, the host selection effect on the fungal community appears to be stronger than that of the bacterial community in adult plants, whereas the opposite is true in the early plant development stages (Bergelson et al., 2019; Zhuang et al., 2020; Xiong et al., 2021b). These clues indicate that variation in the composition and structure of the root endophytic microbiota is non-random. Interestingly, recent studies applying the ecological null modeling framework have confirmed that the assembly of the root endophytic microbiota is primarily affected by stochastic processes (**Table 1**; **Figure 2**) (Birch et al., 2022; Tian et al., 2022; Zhong et al., 2022). Although this finding explains why stochastic assembly dominates the endophytic root microbiota: via priority effects—microorganisms that arrive first at a location have positive or negative effects on those that arrive later—and competitive exclusion, it contradicts the previously established consensus that roots are a highly selective environment that recruits specific microbial taxa via different exudation patterns.

We believe that this explanation is reasonable but unconvincing, and it theoretically supports the compatibility of stochastic dominance and strong host selection. Fungal taxa colonizing the root and soil have little to no overlap, and some specific bacterial taxa are enriched only in the roots (Hu et al., 2020; Zhuang et al., 2020). Indeed, recent studies suggest that the shaping of the community composition of plant rhizocompartments by host selection exceeds the priority effects (Attia et al., 2022). Therefore, priority effects cannot adequately explain the stochastic assembly of the root endophytic microbiota. Furthermore, microbial interactions in root co-occurrence networks tend to have more positive than negative connections (Xiong et al., 2021a). In particular, microorganisms in root endophytic fungal communities generally exhibit cooperative behavior (Kia et al., 2019). In this context, as further discussion is urgently required, we argue that this inconsistency may be related to the current division strategy of the microbial community assembly process, and we focus on the changes in the assembly patterns of root endophytic microbiota when dispersal limitation is attributed to deterministic processes. The results showed a significant increase in the proportion of deterministic processes in some plant taxa previously proven to be dominated by stochastic processes. As a result of this adjustment, the deterministic processes now dominate the assembly of the entire root endophytic microbiota (**Figure 2**). Therefore, the attribution of dispersal limitations is a key factor in determining the relative importance of deterministic and stochastic processes in the assembly of root endophytic microbiota. Importantly, it should be noted that stochastic processes also play a key role in the assembly of root-associated microbiota, especially in microenvironments with reduced host selection pressure (*e.g.*, rhizosphere soil, rhizoplane, and bulk soil) (Gao et al., 2020; Xiong et al., 2021b; Tian et al., 2022). Moreover, functional redundancy resulting from biological interactions and spatial and environmental processes in microbial systems can increase the share of stochastic processes in community assembly. Furthermore, passive recruitment of endophytic microorganisms can occur in plant roots through cracks and be limited by niche occupancy, priority effect, and other events.

## Basis for dispersal limitation subsumption into deterministic processes and suggestions for more rational division in the future

Dispersal refers to the movement and successful establishment of organisms in space and is a basic community assembly process that affects the composition, turnover, and function of the microbial community at evolutionary and ecological levels, and has important implications for improving plant performance and agricultural soil quality (Zhou and Ning, 2017; Choudoir and DeAngelis, 2022; Giovannetti et al., 2023). Factors that lead to low dispersal rates translating to dispersal limitations may be deterministic, stochastic, or both. The new environment has a great influence on the successful establishment of species. In particular, the root endophytic environment has a strong selective effect on the microbiota and manifests only as the enrichment of certain taxa (Hu et al., 2020; Zhuang et al., 2020). These enriched taxa have different dispersal rates depending on their characteristics and active state, which translates into relative importance of dispersal limitation and homogenizing dispersal. Therefore, we propose that dispersal limitation should be attributed to deterministic processes when host selection pressure is exerted on microbiota, as dispersal rates may depend on interactions with the host (**Figure 1**).

Regarding the way microorganisms enter the root environment, host selection of microorganisms has evident chemotaxis characteristics; the host recruits beneficial root endophytic microbiota via specific metabolite signaling (Huang et al., 2019; Koprivova and Kopriva, 2022). Meanwhile, the selected microorganisms also obtain nutritional resources and shelters provided by the plant host (fine root endophytic niches, such as periderm, phloem, and xylem) (Marasco et al., 2022). Therefore, the active migration of microorganisms into the root endophytic environment provides additional evidence that dispersal limitation is more reasonable as a deterministic process. Consistent with this theory, Zhuang et al. (2020) reported that microorganism colonization of mangrove roots is an active process promoted by strong host selection pressure, which intensity may also be a driving force behind the distance decay patterns of the root endophytic microbiota. In this case, the dispersal limitation is also a deterministic process. Compared with endophytic bacterial communities, endophytic fungal communities are more strictly screened by the host adult plant roots and exhibit relatively weaker distance decay (Aas et al., 2019; Zhuang et al., 2020), indicating that host selection and dispersal limitation may have an antagonistic relationship in this case.

More conservatively, in field research related to plant microbiomes in which dispersal traits are difficult to identify, dispersal limitation should be considered as a neutral component rather than a stochastic process, as is the case in most current studies (**Figure 2**). The term “neutral component” refers to an intermediate state between deterministic and stochastic processes, which is neither deterministic nor stochastic or equally divided between the two. It should be noted that in this article, neutral processes follow the unified neutral theory of biodiversity (Hubbell, 2001) equivalent to stochastic processes and are a completely different concept from neutral components. Although this neutralization treatment could not truly reflect the assembly pattern of root endophytic microbiota, it effectively eliminates the community assembly bias resulting from inappropriate attribution of dispersal limitation to stochastic processes.

Collectively, in future research on the assembly mechanism of endophytic root microbiota using the framework of ecological null modeling, we recommend first incorporating dispersal limitation into deterministic processes. In this context, suppose the community assembly patterns (i.e., the proportion of different ecological processes and the relative contributions of deterministic and stochastic processes) do not correspond well to the community convergence, divergence or stochastic variation and their key drivers as revealed by multivariate analyses of endophytic root microbiota (such as non-metric multidimensional scaling, permutational multivariate analysis of variance, and variance partitioning analysis) (Clements, 1916; Gleason, 1926; Zhou and Ning, 2017). In that case, the dispersal limitation should be taken as a neutral component. Further, stochastic processes should be considered when the multiple result patterns mentioned above remain inconsistent in the context of dispersal limitation attributed to neutral components. The level-by-level verification of the attribution of dispersal limitation from deterministic processes to neutral components to stochastic processes may contribute to the final outcomes of root endosphere community structure to be more consistent with the assembly process. Importantly, the neutral community model (Sloan et al., 2006), which divides the entire root endophytic microbiota into three modules (selected, opposed, and neutral taxa) based on 95% confidence intervals, should be closely integrated into the aforementioned level-by-level verification process to roughly estimate the relative contributions of deterministic and stochastic processes (Moroenyane et al., 2021; Zhang et al., 2022), thereby inform the accurate attribution of dispersal limitation and promote the efficient application of methods changing proportions of assembly mechanisms to modify endophytic communities in order to improve crop yield and nutritional quality.

## A potential theoretical framework for dynamic assembly patterns of endophytic microbiota along the soil–plant continuum

**Figure 3:**
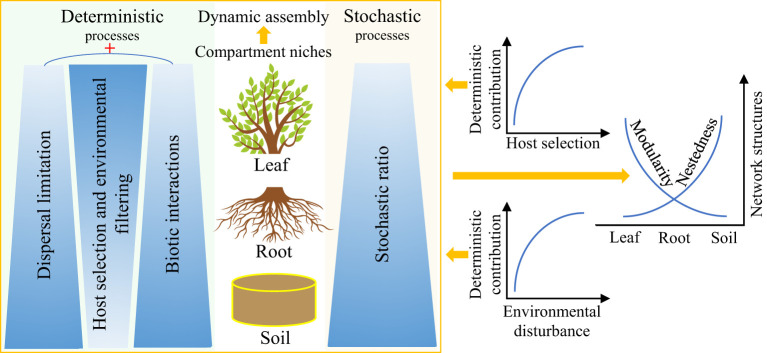
Potential theoretical framework for the dynamic assembly and contrasting co-occurrence patterns of plant endophytic microbiota mediated by compartment niches. Darker colors represent greater contribution of the corresponding ecological process to the assembly of endophytic microbiota along the soil–plant continuum. ‘+‘ represents synergistic effects.

Deterministic and stochastic processes often co-occur in multiple ecosystems and greatly affect the composition, assembly, and function of microbial communities (Zhuang et al., 2020; Liu et al., 2021a). Hydrologic connectivity (including precipitation) and dissimilar plant communities’ composition have been proven to be key regulatory factors of the relative importance of deterministic and stochastic processes (Langenheder and Lindström, 2019; Liu et al., 2021b; Yang et al., 2022). Remarkably, numerous studies have consistently confirmed that compartmentalization along the soil–plant continuum (**Table 1**) is a key driver of plant endophytic microbiota assembly and interactions (Trivedi et al., 2020; Moroenyane et al., 2021; Roux et al., 2023). Herein, we propose a theoretical framework for dynamic assembly patterns of endophytic microbiota along the soil–plant continuum (**Figure 3**). The mutual selection, adaptation, and coevolution of plants and microorganisms can be traced, and the different selection intensities of compartment niches mediate the trade-offs between deterministic and stochastic processes of endophytic microbiota along the soil–plant continuum. From the leaves to the roots and soil, as the selection intensity of the host and environment decreases (Xiong et al., 2021a), the contribution of the deterministic assembly decreases while the stochastic ratio increases.

The synergistic effects of dispersal limitation and biotic interactions enhance the relative importance of the deterministic processes when the intensity of selection by the host decreases. Low dispersal rate, weak selection, and drift can act together to increase the variability or turnover of the soil microbial communities (Zhuang et al., 2020; Bell et al., 2022). In contrast, the endophytic leaf microbiota is strongly influenced by plant phylogeny and usually exhibits a relatively conserved community structure in some wild herbaceous plant species, as well as in plant taxa of different genotypes, possibly as a result of host regulation of keystone taxa performing core functions in the co-occurrence network of the leaf endophytic microbiota (Banerjee et al., 2018; Roman-Reyna et al., 2019; Singer et al., 2019; Massoni et al., 2020; Yang et al., 2023). Furthermore, the deterministic contribution increases with increasing levels of environmental disturbance, which in turn reduces microbial richness, enhances interspecific associations, and organizes communities into relatively tightly linked (i.e., high connectivity) and nested networks with highly asymmetrical interactions (Wang et al., 2021). In addition, the endophytic microbial community exhibits a contrasting network structure along the soil–plant continuum, with nestedness (more specialist species only interact with a subset of the species that interact with the more generalist species) gradually increasing from the leaves to the roots and the bulk soil, whereas modularity decreases (modularity is used to characterize distinct modules of frequently interacting species), with significantly fewer species interacting between these modules than within the modules (Yao et al., 2019; Yang et al., 2023).

Assuming that strong host selection can promote the recruitment of beneficial microorganisms, future research should focus on temporal variation in the assembly patterns of endophytic microbiota at the plant niche level. Previous studies suggested that deterministic processes become increasingly important as the host grows and develops, mainly due to the enhanced host selection filtering the initial microbiota (Gao et al., 2020). Therefore, the period during which the microbiota along the soil–plant continuum (here it refers specifically to bulk soil, root endosphere, and leaf endosphere) experiences the strongest host selection in the plant life cycle can be regarded as a breakthrough for plant microbiome engineering of targeted disease resistance and high yield management, as plants or crops choose the most suitable partners during the strong host selection stage.

In addition, the size-plasticity hypothesis predicts that smaller organisms are less susceptible to environmental and host-specific selection due to their higher metabolic adaptations than larger organisms (Farjalla et al., 2012). Compared to bacteria, fungi have higher carbon use efficiency and can degrade complex substrates and are larger in size (Six et al., 2006). Therefore, the stochastic share of bacterial community assembly may be higher than that of fungal communities. Interestingly, Jiao et al. (2022) revealed that the effect of stochastic processes on bacterial communities decreases with increasing fungal richness. However, how bacteria affect fungal community assembly has been little explored, especially in Streptomycetes growing as pseudomycelia. Recently, the addition of Streptomycetes was found to increase the stochastic share in bacterial community assembly (Li et al., 2022). Furthermore, Streptomycetes addition can promote crop growth and effectively control soil-borne fungal plant pathogens (Li et al., 2019). Therefore, whether and how Streptomycetes affect fungal community assembly is a key question that deserves further exploration and contributes to a deeper understanding of bacterial-fungal interactions.

## Concluding remarks

In some cases, the application of the currently widely accepted community assembly framework in plant microbiomes may not be fully consistent with that in soil, water, and gut microbiomes. Different ecosystems or microenvironments may have unique microbial recruitment or assembly characteristics. Hence, to accurately explain the adaptive significance and functional mechanism of the microbiota under various environmental conditions, we should consider different methodologies, analyze specific situations, and strive to achieve appropriate improvements. We believe that dispersal limitation under strong selection pressure exerted by compartmentalization or host characteristics (such as morphology, structure, immune system, secretions, and genetic networks) and developmental stages should be regarded as a deterministic process, which can better explain the assembly patterns of root endophytic microbiota. Noteworthily, a better understanding on the patterns and convergence of microbiota assembly in different plant and crop lineages is pivotal for the efficient design of resilient synthetic microbial communities and their sustainable application in crop health, high-yield, and high-quality. Taken together, the herein-reviewed perspectives on the root endosphere community structure that cannot be explained by the current framework can help to continually improve and develop this investigation field.

## Data availability statement

The original contributions presented in the study are included in the article/supplementary material. Further inquiries can be directed to the corresponding author.

## Author contributions

JG designed the outline of the manuscript. TL and JG collected data and wrote the manuscript. JG polished the manuscript. All authors contributed to the article and approved the submitted version.
